# Cytotoxicity and Anticancer Activity of *Donkioporiella mellea* on MRC5 (Normal Human Lung) and A549 (Human Lung Carcinoma) Cells Lines

**DOI:** 10.1155/2020/7415672

**Published:** 2020-12-09

**Authors:** Anith M. M. Sairi, Siti Izera Ismail, Arina Sukor, Noraswati M. N. Rashid, Norsazilawati Saad, Syari Jamian, Sumaiyah Abdullah

**Affiliations:** ^1^Department of Plant Protection, Faculty of Agriculture, Universiti Putra Malaysia, 43400 UPM Serdang, Selangor, Malaysia; ^2^Department of Land Management, Faculty of Agriculture, Universiti Putra Malaysia, 43400 UPM Serdang, Selangor, Malaysia; ^3^Agro-Biotechnology Institute Malaysia (ABI) Complex, Jalan Eksotika Off Persiaran MARDI-UPM, Serdang, Seri Kembangan 43400, Selangor, Malaysia

## Abstract

Polypores are mushrooms which are rich in bioactivities and for generations, they have been widely used as herbal remedies. Despite their significant importance in treatments of various health issues, only a few local species have been reported for their pharmacological potentials. The present study was carried out to establish cytotoxicity potentials of *Donkioporiella mellea*, a local polypore species collected from forested areas in Malaysia at cellular levels on normal human lung (MRC5) and human lung carcinoma (A549) cell lines. Survival and inhibition rates were analyzed by 3-(4, 5)-dimethylthiahiazo (-z-y-l)-2,5-diphenyltetrazoliumbromide (MTT) while monitoring changes on cellular shapes by inverted phase contrast microscopy. Survival rates of MRC5 cells were observed to be significantly higher than A549 after treatments with various concentrations of polypore extracts. MRC5 cells showed excellence in survival performance when treated with hot and cold aqueous extracts. Cold aqueous extract showed higher cytotoxicity activities compared to hot aqueous extract (*p* < 0.0001) with inhibitory concentration (IC_50_) values of 414.29 *μ*g/ml and >1000 *μ*g/ml, respectively. Treatments with tamoxifen as a control exhibited necrotic features in both cell lines. The results suggest that *D. mellea* possesses pharmacological potentials that can be utilized for human consumption as a new bioresource alternative, thus encouraging research advancement in mycological and nutraceutical studies.

## 1. Introduction

Malaysia is a megadiverse country with flora and fauna of great biodiversity. Literature has it that tropical climate is the most favorable habitat for diverse species of polypores. Lee et al. [1] listed 4000 species of fungi found mostly from divisions or phyla Ascomycota and Basidiomycota. Estimated at about 70% of macrofungi, these phyla have not been described and discussed [2]. Much of the information and knowledge on the abundance of macrofungi diversity are either outdated or scattered in many different publications and not readily available [2] or already out of print [1].

Numerous Basidiomycetes or commonly known as mushrooms are natural decomposers, which are pathogens or parasites having symbiotic relationships with both animals and plants. Edible mushrooms are always high in worldwide demand as reflected by their annual increase in production [3]. They have unique and subtle savor which made them popular in gourmet cuisines throughout the world [4, 5]. Since the dawn of time, besides having good flavor favored by many, mushrooms have also been, for centuries, routinely used as remedies by indigenous Chinese. Abundant bioactive compounds with antioxidants properties have been widely cited [6–8]. Europe, five popularly known polypore mushrooms, sometimes known as European ancestors' heritage, include *Laetiporus sulphureus, Fomes fomentarius, Fomitopsis pinicola, Piptoporus betulinus,* and *Laricifomes officinalis* [9]. In Asian countries such as Japan, Korea, and China, the species *Phellinus linteus* has been regarded as a traditional Chinese medicine with a 2000-year-long history of being used in medicinal applications for treatments of hemorrhages, hemostasis, and disease related to female menstruation [10, 11]. Several medicinal species of mushrooms endowed with medicinal properties such as *Ganoderma lucidum, Lignosus rhinocerotis* (tiger milk mushroom), *Lentinus* spp.*, Hericium erinaceum* (monkey head mushroom), *Termitomyces* spp., *Schizophyllum* spp. and others have also been well documented. However, evaluations on their pharmacological potentials are still advancing with some exhibiting high market prospects.

Natural products from biological organisms have served as favorable and affordable sources for new drug entities. These natural products have led to the development of numerous vaccines and other medicinal products [12]. However, some organisms, including mushrooms, are known to be poisonous to humans and animals, by virtue of the organisms' natural survival and defense system to sustain in the environment. Against this background, cytotoxicity study is important as a primary step in determining the potentials of biologically active compounds isolated from these organisms [13]. In the pharmaceutical or cosmetic industries, cytotoxicity study is considered a standard of procedure to establish toxicity level of any new drugs or products. Successful development of new discovery is dependent on these toxicity results, either minimal or nontoxic. In this regard, studies in cellular toxicity play a crucial role as a way of introducing new variety of edible organisms which are safe for human consumption and have the potential of exploiting their therapeutic components.

Edibility of mushrooms is based on such criteria as being nonpoisonous, desirable taste and aroma [14], and unique texture [15]. These mushrooms are known as culinary mushrooms [16]. Edible mushrooms are mainly consumed for their nutritional or dietary benefits. However, advances in research have discovered that mushrooms have metabolites that are medically significant and have been utilized for their anticancer, antimicrobial, antioxidant, immunomodulatory, antihypersensitive [17, 18], antiatherosclerotic, and anticarcinogenic properties [19, 20]. For this reason, edible mushrooms are those that can be consumed as either culinary-medicinal mushrooms or edible-medicinal mushrooms [16]. However, not all medicinal mushrooms are readily edible due to hard texture of their fruiting bodies. These types of mushrooms are consumed in the form of powdered extract. There are several poisonous mushrooms that had been reported such as *Entoloma rhodopolium* and *Scleroderma citrinum* which are known to cause rhabdomyolysis and gastrointestinal distress in animals and humans [16, 21]. The primary purpose of the present study was to introduce a new species of local polypore mushroom establishing its cytotoxic efficacy at the cellular level with a view of promoting further research and development of mushrooms research advances in Malaysia.

## 2. Materials and Methods

### 2.1. Reagents

Reagents used in the present study included the following: RPMI 1640 (Rosewell Park Memorial Institute) medium (from Gibco), phosphate buffer saline (from PBS-Sigma), Pen-strep (Penicillin–Streptomycin Antibiotic, (from Gibco), fungizone (from Gibco), fetus bovine serum (from FBS-Gibco), MTT (3-(4, 5)-dimethylthiahiazo (-z-y-l)-2, 5-diphenyltetrazoliumbromide), DMSO (Dimethyl Sulfoxide), Tamoxifen, and trypsin with phenolic red (with 0.05–0.25%) (from Gibco).

### 2.2. Sample Preparation and Mycelium Accumulation

Polypore mushroom specimens were collected from forested areas in Kepong, Selangor, Malaysia in April 2018 during a dry season. The sample was isolated for pure culture and deposited at the Agro-Biotechnology Institute (ABI) collection deposit centre with a voucher code ABI002. The mushroom species, *Donkioporiella mellea*, was identified by mycologist (ABI) through morphological and molecular methods. The sequences obtained from the specimens were submitted to a GenBank database, an open access, annotated collection of all publicly available nucleotide sequences and their protein translations. The accession number was MT229067. Ten plugs of mycelia from agar culture were transferred into flasks containing 100 ml of mushroom complete medium (MCM) with some modifications. The flasks were placed on a rotary shaker and run at 150 rpm at 28°C for 14 days. Subsequently, the mycelia and culture broth were harvested and kept in a −80°C freezer overnight and dried by freeze-drying (Labconco freeze-dry machine) for 3 days. Mycelium biomass were mashed into fine powder for further extraction process.

### 2.3. Preparation of Extract

In the present study, the extraction procedures used were by hot and cold aqueous extraction methods. Double-distilled water was used as a solvent throughout the extraction processes.

#### 2.3.1. Hot Aqueous Extraction

The extraction method procedure was adopted from a previous study by Lee et al. [22] with some modifications. Ten grams of mycelium powder were boiled in a 200 ml of double-distilled water in a ratio of 1 : 20 with temperature ranging from 90°C to 95°C for 20 to 30 minutes. The samples were subsequently centrifuged at 8000 × g for 15 minutes and the supernatant was filtered using Whatman No.1 filter paper. The residues were reextracted with 100 ml of distilled water. The filtrate from two rounds of extractions were combined and dried by freeze-drying. The extract obtained and stored at 4°C was used directly for evaluation of cytotoxicity.

#### 2.3.2. Cold Aqueous Extraction

The extraction method for cold aqueous extract was adapted from Tseng and Mau [23]. The ratio of mycelium sample to double-distilled water was 1 : 10. Ten grams of mycelium powder was added to100 ml of distilled water in a 500 ml flask while shaking vigorously. The flask containing the sample mixture was let to stand while stirring at 100 rpm for 24 hours at room temperature (25°C). Subsequently, the mixture was centrifuged at 5000 × g for 15 minutes and the supernatant was filtered through Whatman No. 1 filter paper. The residue was reextracted by adding 100 ml of distilled water. The filtrates from two rounds of extractions were combined and freeze-dried. The extract obtained was stored at 4°C and used directly for cytotoxicity evaluation.

### 2.4. Cell Viability

#### 2.4.1. Cell Culture

Cell culture and assay protocols were conducted in a biosafety cabinet Class 2. Two types of cell lines were used: MRC5 (normal human lung fibroblast) and carcinoma human lung epithelial (A549) previously obtained from American Type Culture Collection (ATCC). These cells were cultured in RPMI-1640 (Rosewell Park Memorial Institute) medium supplemented with 10% of fetal bovine serum (FBS), 1% of pen-strep (penicillin streptomycin), and 0.5% of fungizone. Both cells types were cultured in an incubator set at 37°C and humidified with 5% of CO_2_ atmosphere. All cells were cultured in 2-day intervals. Growth responses were observed on a daily basis under an inverted microscope. Only cells at their exponential growth were used throughout the experiments.

#### 2.4.2. Treatment Application

Solutions of fresh extract were prepared by dissolving extracts of *D. mellea* with sterile distilled water while tamoxifen was used as a control. All mixtures of extracts were filtered using 0.22 nm filter membrane before transferring them into 96-well plates containing the two cell lines. Precisely, an amount 1 × 10^4^ of cell concentrations were seeded in each well for 24 hours before treatment application of treatments. Cells in each of the 96-well plate were treated with different concentrations gradient of samples mixture from 1.95 *μ*g/ml to 1 mg/ml for different times of exposure (24, 48, and 72 hours). The procedure was done in three replications.

#### 2.4.3. MTT Assay

Cell viability was determined through MTT bioassay protocols adopted from Mosmann [24]. Following incubation in different concentrations of samples, an amount of 15 mg of MTT powder was weighed and diluted in 3 ml of phosphate-buffered saline (PBS) solution to obtain a concentration of 5 mg/ml. The MTT solution was filtered using 0.22 nm filter membrane into a Petri dish. Treated plates were retrieved from the incubator and 20 *µ*l MTT solution was pipetted into each well. The plates were reincubated in an incubator for 4 hours after which the solution was discarded leaving only the purple formazan crystals. An amount of 100 *µ*l DMSO was added into all wells to dissolve the purple formazan crystals by gently resuspending until completely dissolved. Absorbance of the assay was measured using a microplate reader at 590 nm.

#### 2.4.4. Parameter

Cell performances after treatment were evaluated through proliferation and inhibition rates of the cells. Data were taken at 24-, 48-, and 72-hour intervals. Both proliferation and inhibition parameters were obtained from MTT assay results, based on the following equations:

*(1) The proliferation rate of cells (%)*

(1) $$\begin{array}{c} \frac{\left(AT-AB\right)}{\left(AC-AB\right)}\times 100\mathrm{\%,} \end{array}$$

where AT is the  absorbance of treated cells, AB is the absorbance of blank (media only), and AC is the absorbance of control (untreated cells).

*(2) The Inhibition rate of cells or cytotoxicity (%):* = 100 −  total number of survived cells (%).

#### 2.4.5. Statistical Analysis

All results of the study were expressed as means ± standard error of mean (SEM). Significance differences were evaluated by Analysis of Variance (ANOVA). A probability level of *p* < 0.05 was considered statistically significant. All data were analyzed using SAS (Statistical Analysis System) software version 9.4.

## 3. Results and Discussion

### 3.1. Toxicity Effect of Different Extracts at Cellular Levels

**Figure 1:**
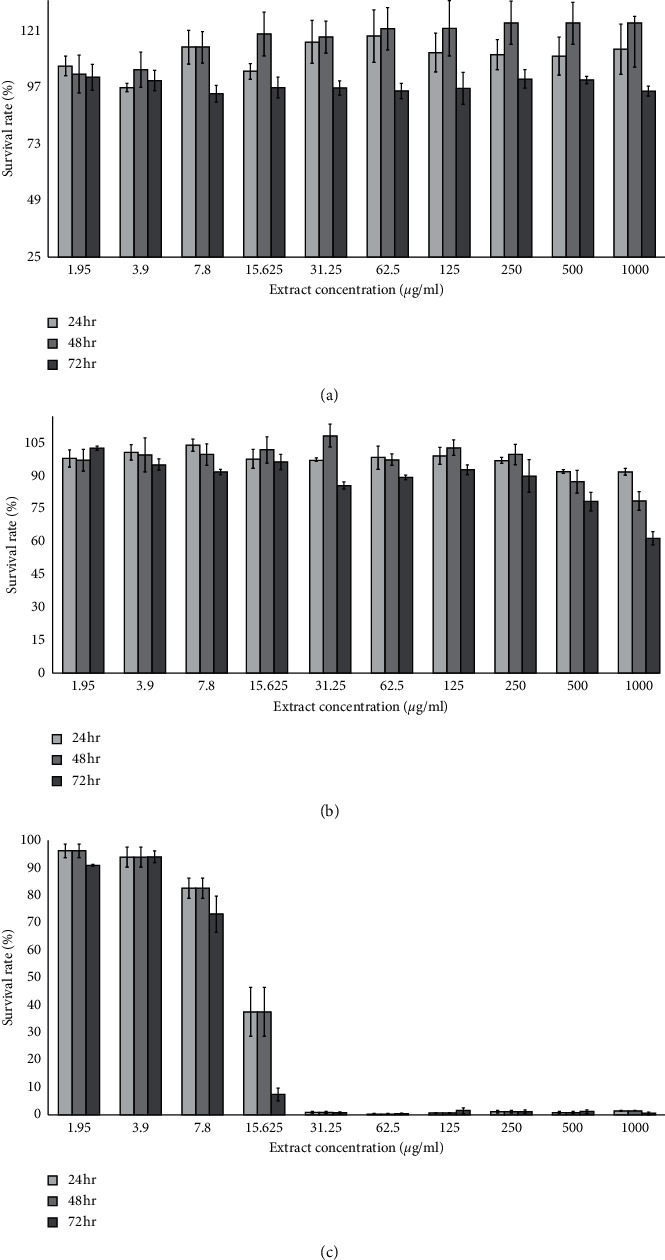
Inhibition effects on MRC5 cells after 24, 48, and 72 hr of time exposure measured by MTT. (a) Survival rates of MRC5 cells treated with different concentrations of hot aqueous extract. (b) Survival rates of MRC5 cells treated with different concentrations of cold aqueous extract. (c) Survival rates of MRC5 cells treated with different concentrations of tamoxifen as control.

The proliferation rate of MRC5 cell line treated with hot aqueous extract of *D. mellea* showed no significant changes (*p* < 0.05) as the concentrations were increased. The survival rate was significantly higher, as treatment application times were prolonged up to 72 hr as presented in Table 1. On the contrary, treatment with cold aqueous extract of *D. mellea* showed acute cell inhibition at the highest concentration compared to hot aqueous extract with rate of inhibition being less than 40% after 72 hr of treatment (Figure 1). However, the IC_50_ value for both *D. mellea* extracts were more than 1000 *μ*g/ml on MRC5 cell line, suggesting that this mushroom was safe for human consumption. Tamoxifen was used as a positive control. The characteristics of tamoxifen were toxic at cell levels showing lowest survival rate among other treatments in a dose-dependent manner (Figure 1). Although tamoxifen was harmful to MRC5 cell line, the cells still survived at lower concentrations (1.95, 3.9, 7.8, and 15.625 *µ*g/ml) with survival percentages of 94.4, 93.8, 79.4, and 27.5%, respectively. The survival rates for other concentrations were approximately zero. Cell survival rates for other concentrations were recorded to be near zero. As presented in Figure 1, the stability of the reaction between extracts and cells gave an indication of being time-dependent. In some treatments, survival rate slightly increased from 24 to 48 hours of exposure. Exposure for 72 hours recorded significant changes for all treatments, suggesting that exposure at longer time was needed for maximum damage, which, in turn, influenced the stability of samples, and reactions of cells.

**Figure 2:**
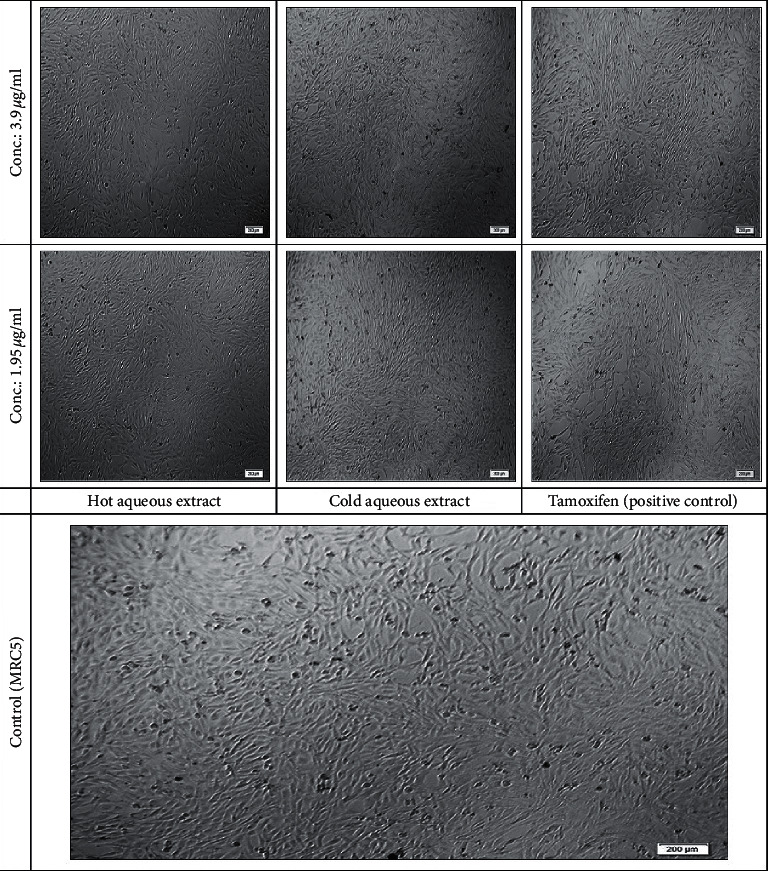
Morphological changes of MRC5 cell after 72 hr treatments induced by hot aqueous extract, cold aqueous extract, and tamoxifen as positive control in concentrations (conc.) of 1.95 to 3.9 *µ*g/ml observed under inverted phase contrast microscopy (20x total magnification). Scale bar: 200 *µ*m.

Under an inverted microscope, changes in the morphological phase were observed for different treatments after 72 hr of exposure. Normal cells of MRC5 (Figure 2) were observed as fibroblast cells flat, elongated, and spindle-shaped (aligned in parallel cluster). The application of hot extract of *D. mellea* did not trigger any morphological changes on the cells. The proliferation rate of cells as well as untreated MRC5 cell growth was satisfactory. For cold extract of *D. mellea* treatment, as the gradient concentrations changed (except at 1000 *μ*g/ml concentration), the cells became thickened and round in shape, but adherent ability remained. Tamoxifen application caused significant variations in cell morphology in a dose-dependent manner where higher doses caused cell lysis and dead cells floating in the medium.

**Figure 3:**
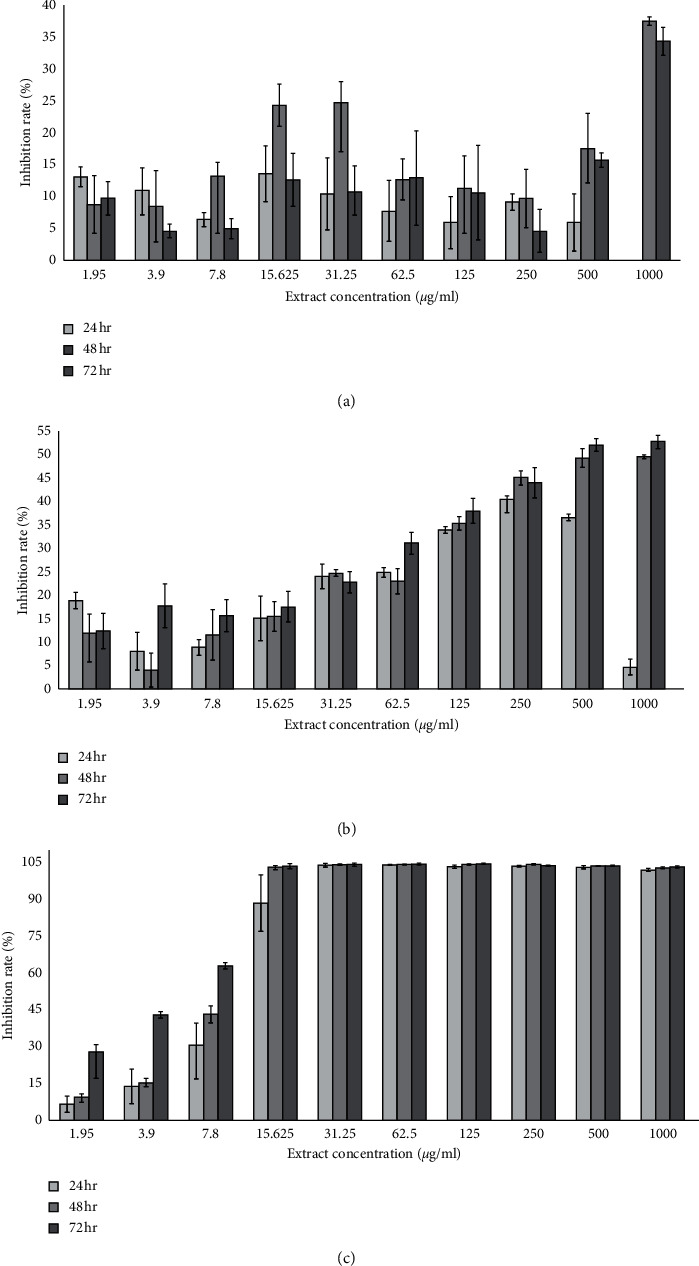
Cytotoxicity effects on A549 cell lines after 24, 48, and 72 hr measured by MTT. (a) Inhibition rates of A549 cells treated with different concentrations of hot aqueous extract. (b) Inhibition rates of A549 cells treated with different concentrations of cold aqueous extract. (c) Inhibition rate of A549 cells treated with different concentrations of tamoxifen as control.

A549 cell line showed lethal effects for all extracts' types of varying concentrations with variations in survival rates. The present study observed that proliferation rate of A549 cell line decreased in a time-dependent manner such that as time duration increased, inhibition rate increased. Cells treated with hot aqueous extract of *D. mellea* were observed to have slower changes in proliferation rate as concentrations were increased. Significant toxicity effect was observed in time-dependent manner, the highest being at the highest concentration (1000 *μ*g/ml) of hot aqueous extract, suggesting that growth of cancer cells could be inhibited by more than 30% as time was extended using hot aqueous mycelium extract of *D. mellea* at 1000 *μ*g/ml concentration. However, the survival rate of A549 cell line was significantly affected by cold aqueous compared to hot aqueous extract. The proliferation rate of A549 significantly decreased at higher concentrations (500 and 1000 *μ*g/ml) in a time-dependent manner as shown in Figure 3. The lethal effect of tamoxifen can be seen clearly on how the cells reacted towards different dosages of tamoxifen. Tamoxifen killed the cells directly and effectively compared to *D. mellea* extracts. As shown in Table 2, the IC_50_ value of tamoxifen was lower than both extracts of *D. mellea*. Cell survival rate decreased as concentration increased across all extracts applications.

**Figure 4:**
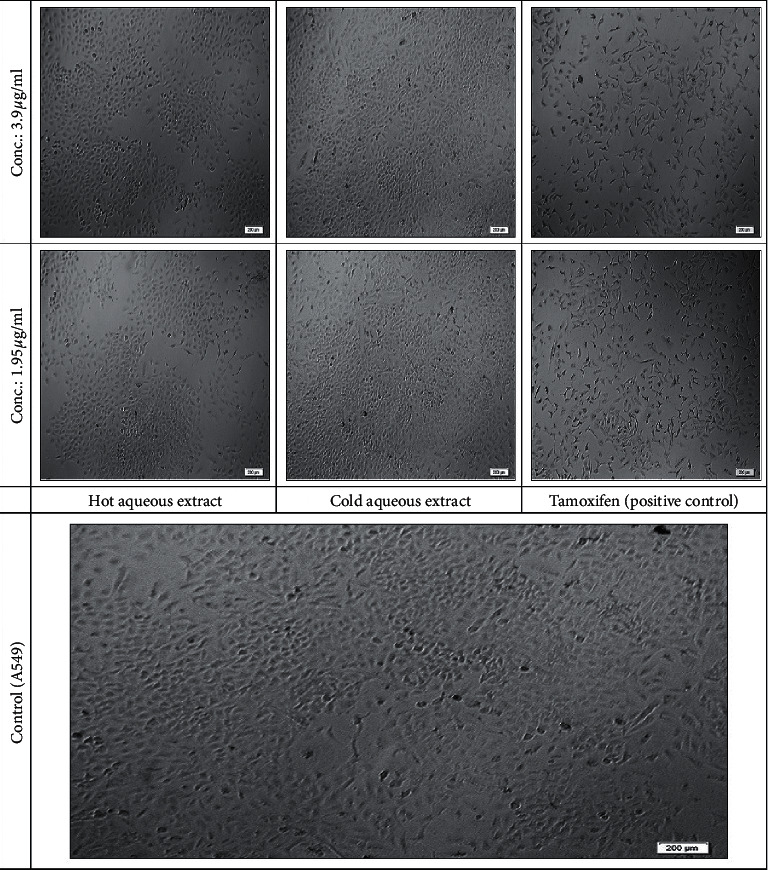
Morphological changes in A549 cells after 72 hr treatments induced by hot aqueous extract, cold aqueous extract, and tamoxifen as positive control in concentrations (conc.) of 1.95 and 3.9 *µ*g/ml observed under inverted phase contrast microscopy (20x total magnification). Scale bar: 200 *µ*m.

Untreated A549 cell line had polygonal shape and remained proliferating to a confluent level (Figure 4). On the other hand, cells treated with different concentrations of both aqueous extracts of *D. mellea* induced obvious morphological changes at the highest concentration. Hot water extract showed some symptoms in separation of cell fragments and in density at 1000 *µ*g/ml. Cell density decreased in treatment with 500 *µ*g/ml as compared to the untreated (control). At similar concentrations (500 and 1000 *µ*g/ml) for cold aqueous extract, the cells progressively shrunk to smaller, rounded shape and some of the cells were observed to be detached. However, at higher concentrations, the separation of cell fragments was observed on cells treated with cold water aqueous compared to hot aqueous extract treatment. At lower concentrations, most cells remained in elongated shape with adherent ability and still attached to the surface of flasks. The majority of A549 cells were completely detrimental, remained detached, and floated in the medium after treatment with tamoxifen at concentrations of 15.625 to 1000 *µ*g/ml. Tamoxifen at concentration lower than 15.625 *µ*g/ml caused changes in cell density.

In treatments of less than 72 hr duration, the effects were clearly seen on proliferation and inhibition rates of MRC5 where maximum damage occurred in the cells. The cells responded differently according to treatments. Changes in the effects of cytotoxicity as recorded in the study suggest that MRC5 cells were a toxicity marker at cellular level, as well as an indicator of whether the mushroom was edible and safe for human consumption. The present study also observed that hot aqueous mycelium extract of *D. mellea* was safe for human consumption as the proliferation rate was recorded to be consistent (Figure 1). As concentrations increased, survival rates remained high. However, cold aqueous mycelium extract of *D. mellea* demonstrated low selection ability with acute cytotoxicity at higher concentration (61.3% at 1000 *µ*g/ml) (Figure 1) at which the extract inhibited normal lung cells (MRC5) with 38.7%. Hot aqueous extract at lower concentrations showed cytostatic effect on A549 cell line at lower concentration but became cytotoxic at the highest concentration. Cold aqueous extract of *D. mellea* showed higher cytotoxicity effect compared to hot aqueous extract. For hot and cold aqueous extracts of *D. mellea*, cell inhibition of A549 increased as treatment duration was extended. Both extracts showed significant difference (*p* < 0.05) at higher concentrations of aqueous extract of *D. mellea* against A549 cell line. At lower concentration (15.25 *µ*g/ml), tamoxifen yielded necrotic symptoms on MRC5 and A549. Tamoxifen produced the highest rate of inhibition compared to the two different aqueous extracts of *D. mellea*.

As the concentrations were increased, hot aqueous extract induced inhibition effects at moderate level on A549. The findings suggest that hot aqueous extract had low anticancer effect (IC_50_ > 1000 *µ*g/ml), possibly due to different pharmacological potential as reported in numerous other studies. Hot aqueous extracts of mushroom have been reported to have copious amount of carbohydrate and protein as high-molecular-weight polysaccharides and polysaccharide-protein complexes but low in fat. This feature sets mushroom as immunomodulatory by modulating immune system in the human body rather than targeting cells directly [25, 26]. Lai et al. [27] reported that the hot water extract of *Polyporus rhinocerus* exhibited moderate cytotoxicity against HL-60 (IC_50_∼1000 *µ*g/ml). The reports concurred with Lau et al. [28] who also reported that hot aqueous extract of *Lignosus rhinocerotis* reduced viability of several cancer cells (A549, MCF7, HL-60, PC3, and HK1) by 50% after 72 hr duration of treatment exposure at higher concentration. Hot aqueous extracts of mushrooms were proven to be less effective against tumor cells directly unless specific extraction protocol was carried out. Jamil et al. [26] reported that boiled water extract from tuber of *L. rhinocerotis* had mild cytotoxicity activity against A549 while purification of the extract changed the cytotoxicity level (57.78 *µ*g/ml) of the tuber on the same cell type.

Cold aqueous extract was observed to have good antiproliferation effect on A549 compared to hot aqueous extract with an IC_50_ value of 414.29 *µ*g/ml (Table 2). The finding was in line with several studies with hispolon-induced apoptosis in lung cancer cells A549 and H66 but different in their levels of cytotoxicity. Ethanol extract from fruiting bodies of *Phellinus igniarius* had IC_50_ value of 531.7 *µ*g/ml against A549 but had lower cytotoxicity against SGC-7901 (gastric cancer) with an IC_50_ value of 110.7 *µ*g/ml [29]. In contrast with previous discoveries, *D. mellea* extract tested in the present study had lower IC_50_ value suggesting higher level of cytotoxicity activity against A549 cells compared to *P. igniarius*. However, each mushroom has specific ability against specific tumor cells depending on its bioactive constituents. As an example, total triterpenes of *G. lucidum* were reported to be highly effective in inhibiting proliferation of MCF7 cells at concentration of 100 *µ*g/ml [30] while polysaccharide extract of the same had immunomodulatory activity [31]. These conflicting findings were likely due to differences in the extraction methods and solvents used in the extraction protocol as differences in chemical composition yielded different outcomes. Nevertheless, the ability of cold aqueous mycelium extract against A549 cell was slower compared to other mushroom types which could be related to bioreactivity at cellular level from different extraction methods and parts of mushrooms sourced. As proven by several other studies, extracts from different parts of mushrooms (such as vegetative part) and harvest stages (different physiological stages) were reported to have different levels of bioactivities [32, 33]. Various cytotoxicity levels in mushrooms against A549 cells have been reported from various sources and extract types such as polysaccharide extract of *F. fomentarius* from fruiting bodies (IC_50_ : 100 *µ*g/ml), ethanol extract of sclerotia of *Poria cocos* (IC_50_ : 301.1 *µ*g/ml), and aqueous extract of *L. rhinocerotis* (IC_50_ : 41.13 *µ*g/ml) [28, 34, 35].

The cytotoxic effect of aqueous extracts of *D. mellea* affected morphological structures of A549 cells in a concentration-dependent manner after 72 hr of treatment exposure. At higher concentration of cold aqueous application, A549 cell line became refractile under microscopic observations as the cells progressively shrunk to smaller, rounder shape and started to detach. Some of dead cells were observed to become detached and floated in the medium after treatment of cold aqueous at concentrations of 500 and 1000 *μ*g/ml. In hot aqueous extract, the changes occurred at the highest concentration (1000 *μ*g/ml) tested. MRC5 cells showed insensitive reactions to hot aqueous extract of *D. mellea*. The extract triggered proliferation rate at the highest confluent level, causing cytotoxicity effect on A549 cells. Selective ability of hot aqueous extract could be related to inhibition of mitochondrial Complex 1, where a class of therapeutic drugs like lipophilic compounds tended to inhibit mitochondrial bioenergetics, mitochondrial respiration in tumour cells at relatively nontoxic concentrations [36]. Changes in morphological appearance of cells observed as cell death were a reliable basis indicator that cells were undergoing process of apoptosis [37]. This apoptosis is a physiological program which eliminates cells by disrupting morphological and physiological characteristics of cells shrinkage, chromatin condensation, internucleosomal DNA fragmentation, and formation of apoptotic bodies. This has been an approach used in establishing cytotoxic effects of most anticancer agents [30, 38, 39].

## 4. Conclusions

The present study concludes that hot aqueous extract of *D. mellea* was sufficiently safe for human consumption at the cellular level. Hot aqueous extract had shown acute anticancer effects at a concentration of 1000 *μ*g/ml. Cold aqueous extract of the fungus had anticancer potential as shown by its significant cytotoxic effect against A549 cells. The study shows that MRC5 cells had excellent survival performance when treated with hot and cold aqueous extracts. Further investigation through *in vivo* cytotoxicity studies, which will validate the edibility and toxicity of this mushroom before being used for human consumption, is particularly crucial. The exploration effort at further unveiling the capability of *D. mellea* is strongly recommended. The anticancer capabilities of *D. mellea* need to be further investigated in various cancer types and stages, exploiting the mushrooms' full potential as a new alternative in pharmacological and medicinal applications.

## Figures and Tables

**Table 1:** Effects of time of exposures (24, 48, and 72 hours) and different types of extracts (hot aqueous, cold aqueous, and tamoxifen) on IC_50_ values for cytotoxicity of MRC5 cell lines. Cytotoxicity of extracts on A549.

Times of exposure (hour)	Types of extracts (*μ*g/ml)		
	Hot aqueous	Cold aqueous	Tamoxifen
	IC_50_ (*μ*g/ml)	IC_50_ (*μ*g/ml)	IC_50_ (*μ*g/ml)
24	>1000	>1000	21.42
48	>1000	>1000	14.29
72	>1000	>1000	10.0

**Table 2:** Effects of times of exposure (24, 48, and 72 hours) and different types of extracts (hot aqueous, cold aqueous, and tamoxifen) on IC_50_ values for cytotoxicity of A549 cell lines.

Times of exposure (hour)	Extracts type (*μ*g/ml)		
	Hot aqueous	Cold aqueous	Tamoxifen
	IC_50_ (*μ*g/ml)	IC_50_ (*μ*g/ml)	IC_50_ (*μ*g/ml)
24	>1000	>1000	11.42
48	>1000	>1000	7.14
72	>1000	414.29	4.2

## Data Availability

All data used to support the findings of the present study are included within the article.
